# Application of “B+1” heterologous boosting strategy for preventing infection of SARS-CoV-2 variants with resistance to broad-spectrum coronavirus vaccines

**DOI:** 10.1080/22221751.2023.2192817

**Published:** 2023-04-03

**Authors:** Zezhong Liu, Lu Lu, Shibo Jiang

**Affiliations:** a Key Laboratory of Medical Molecular Virology (MOE/NHC/CAMS), Shanghai Institute of Infectious Disease and Biosecurity, School of Basic Medical Sciences, School of Pharmacy, Shanghai Medical College, Fudan University, Shanghai, People’s Republic of China

First-generation SARS-CoV-2 vaccines based on different platforms have significantly reduced hospitalization and death. However, the constant evolution of SARS-CoV-2 has only prolonged the global pandemic. Recent emergence of the Omicron subvariants XBB and BQ.1.1 has posed an unprecedented challenge to the efficacy of current broad-spectrum SARS-CoV-2 vaccines. Several lines of evidence have demonstrated that the majority of the therapeutic monoclonal neutralizing antibodies (NAbs) lost their activities against XBB and BQ.1.1 [1,2]. Dramatic decline of the neutralizing antibody titre against XBB and BQ.1.1 was founded in the sera from vaccinees and infected persons [2,3]. Some SARS-CoV-2 Omicron subvariants, such as XBB and BQ.1.1, are even more resistant than SARS-CoV to NAbs elicited by SARS-CoV-2 ancestral strain, although there are about 50 different amino acids between RBDs of SARS-CoV-2 ancestral strain and SARS-CoV, and 21 different amino acids between RBDs of SARS-CoV-2 ancestral strain and BQ.1.1, suggesting that the global pandemic has remarkably promoted the immune escape mutations of some Omicron subvariants. This calls for the development of a novel immunization strategy to prevent infection from dominantly circulating SARS-CoV-2 variants with exceptional resistance to neutralizing antibodies elicited by broad-spectrum vaccines, such as XBB and BQ.1.1.

In 2008, Lu S and colleagues first used a heterologous prime-boost vaccination regimen to evaluate the efficacy of an influenza virus vaccine. They used an influenza HA-based DNA vaccine and an inactivated influenza vaccine for prime and boost, respectively. This regimen was found to be more effective than the homologous prime-boost regimen using DNA or inactivated vaccine alone to induce antibody responses against H1 or H3 serotype influenza viruses [4]. This strategy was also applied in vaccination against other pathogens, such as HIV, HSV-2, *Plasmodium falciparum,* and *Mycobacterium bovis* [5].

Most recently, heterologous prime-boosting has been successfully applied in vaccination against infection of SARS-CoV-2 and its variants. For example, in one recent study, individuals were primed with two doses of inactivated SARS-CoV-2 vaccine (CoronaVac). Then, 3–9 months later, these subjects were boosted with orally aerosolized adenovirus type-5 vector-based COVID-19 vaccine (Ad5-nCoV). The sera were collected from individuals about 12 months after heterologous boosting and their NAb geometric mean titres (GMTs) were determined. They were found to be 26-fold higher than those of the control group primed with two-dose CoronaVac and boosted with one-dose CoronaVac against authentic wild-type SARS-CoV-2. The orally aerosolized Ad5-nCoV is supposed to be able to elicit respiratory mucosal immunity such as secretory IgA, which would play an important role in preventing viral transmission. These results confirmed that heterologous boosting is more effective in eliciting NAb responses than the homologous boosting regimen [6].

**Figure 1.**
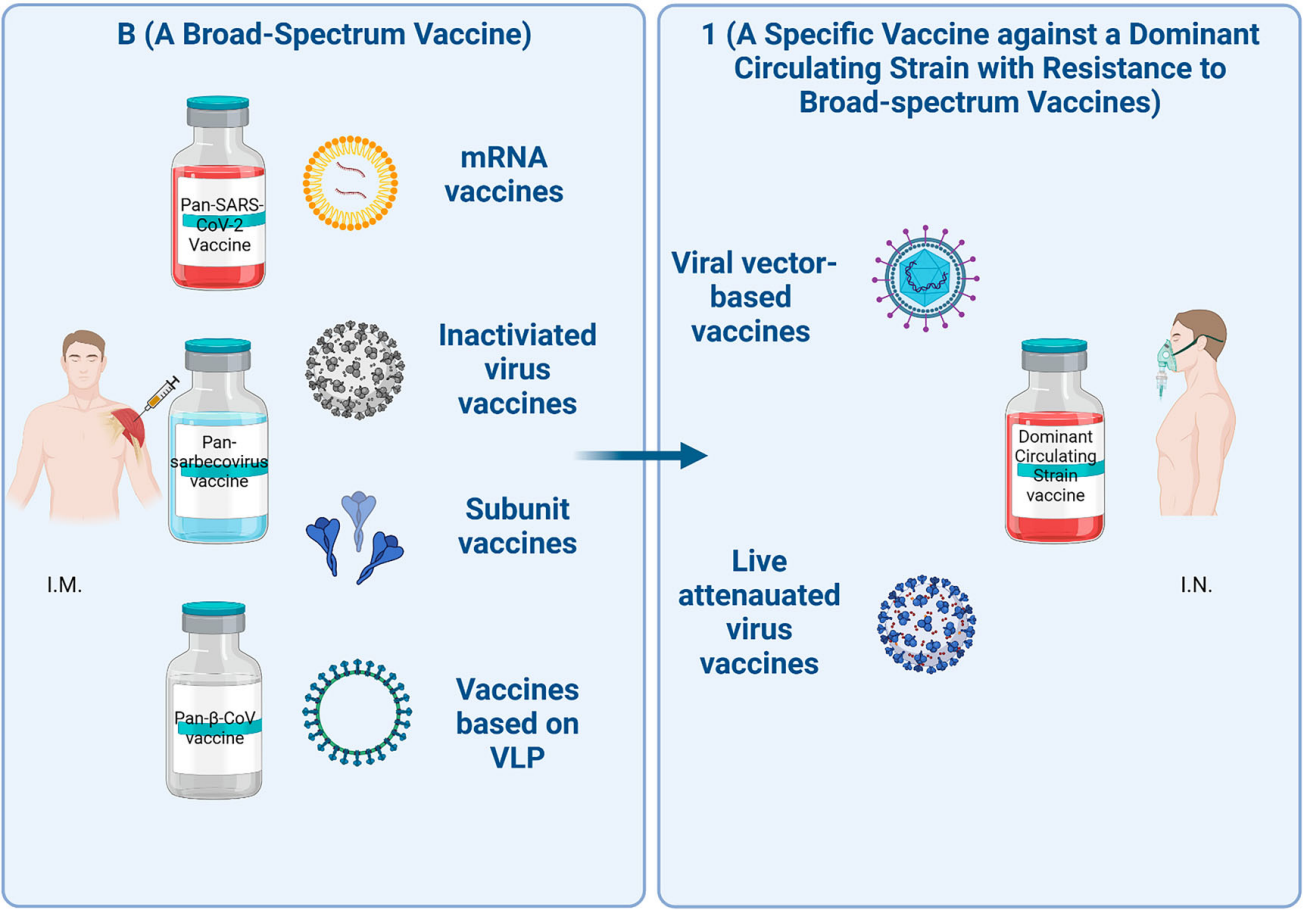
“B+1” heterologous boost vaccination strategy. Individuals will be immunized with a broad-spectrum coronavirus vaccine, such as pan-SARS-CoV-2 vaccine, pan-sarbecovirus vaccine, or pan-β-CoV vaccine via intramuscular route (I.M.), followed by heterologous boosting immunization using a vaccine specific against a dominant circulating viral variant with exceptional resistance to NAbs elicited by broad-spectrum vaccines via intranasal route (I.N.), such as XBB or BQ.1.1.

Encouraged by these findings, we propose a “B+1” heterologous boosting vaccination strategy whereby a broad-spectrum vaccine, denoted as “B” for prime immunization, and a specific vaccine against a dominant circulating variant with exceptional resistance to NAbs elicited by a broad-spectrum vaccine, such as XBB or BQ.1.1, denoted as “1” for heterologous boosting (Figure 1). The “B” and “1” vaccines can be produced from the same, or different, vaccine developers.

Some broad-spectrum vaccines have been developed to address the evolution of SARS-CoV-2 variants. Notably, the updated bivalent mRNA (encoding S proteins of SARS-CoV-2 wild-type strain and BA.5 Omicron variant) vaccine was licenced by the U.S Food and Drug Administration in an effort to stem the tide of BA.5 infections and reinfections [7]. Moreover, some pan-SARS-CoV-2, pan-sarbecovirus, and pan-β-CoV vaccines are under development. For example, the novel STING agonist CF501-adjuvanted RBD-Fc vaccine could elicit cross-NAbs against several SARS-CoV-2 variants, SARS-CoV and bat SARS-related coronaviruses (SARSr-CoVs) [8,9]. Some nanoparticles, such as SpyCatcher003-mi3, I53-50A/I53-50B, or ferritin, were used to display multiple RBDs from different β-CoVs and induce broad protective immunity [10]. Vaccines targeting immutable epitopes, such as HR121, which contains HR1-linker1-HR2-linker2-HR1, could also elicit cross-NAbs against SARS-CoV-2 variants [11]. Finally, the vaccine containing a pan-vaccine antigen (S_pan_) designed based on phylogenetic analysis could elicit broadly potent NAbs against SARS-CoV-2 variants, as well [12]. Apart from the above strategies to improve the broad-spectrum immunogenicity, development of appropriate delivery systems for the vaccine and adjuvant with good safety is needed.

However, some SARS-CoV-2 Omicron subvariants, such as XBB and BQ.1.1, are extremely resistant to NAbs elicited by some broad-spectrum vaccines, including the updated Moderna bivalent mRNA vaccine [3], finally becoming the dominant strain circulating worldwide. To combat this mutational trend, a specific vaccine against a dominant circulating strain with resistance to the broad-spectrum vaccines could be promptly developed using the rapid vaccine development platforms and applied for heterologous boosting vaccination after prime immunization with a broad-spectrum vaccine, as herein suggested.

In summary, our “B+1” heterologous strategy first emphasizes the development of broad-spectrum vaccines to combat current and future emerging SARS-CoV-2 variants, as well as other sarbecoviruses. This important first step is supplemented with the development of a dominant circulating strain-specific vaccine used as a booster at a specified interval. This strategy is specifically designed to address the growing number of subvariants with exceptional resistance to NAbs elicited by broad-spectrum vaccines. The “B+1” strategy can also be used to control outbreaks caused by other highly variable viruses.
